# A 120-second stretch improves postural control and plantar pressure: quasi-experimental study

**DOI:** 10.1590/1516-3180.2021.0255.23072021

**Published:** 2022-03-18

**Authors:** Eva María Martínez-Jiménez, Marta Elena Losa-Iglesias, Sara González-Martín, Daniel López-López, Andrea Roca-Dols, David Rodriguez-Sanz, Ricardo Becerro-de-Bengoa-Vallejo, César Calvo-Lobo

**Affiliations:** I PD, PT, MSc, PhD. Assistant Professor, Facultad de Enfermería, Fisioterapia y Podología, Universidad Complutense de Madrid, Madrid, Spain.; II PD, MSc, PhD. Full Professor, Faculty of Health Sciences, Universidad Rey Juan Carlos, Alcorcón, Spain.; III DN, MSc. Researcher, Facultad de Enfermería, Fisioterapia y Podología, Universidad Complutense de Madrid, Madrid, Spain.; IV PD, MSc, PhD. Senior Lecturer and Researcher, Health and Podiatry Group, Department of Health Sciences, Faculty of Nursing and Podiatry, Universidade da Coruña, Industrial Campus of Ferrol, Spain.; V PD, MSc, PhD. Researcher, Faculty of Health Sciences, Universidad Rey Juan Carlos, Alcorcón, Spain.; VI DT, MSc. PhD. Senior Lecturer, Facultad de Enfermería, Fisioterapia y Podología, Universidad Complutense de Madrid, Madrid, Spain.; VII RN, BSc, MLIS, DPM, DHL, PhD, FFPM, RCPS. Full Professor, Facultad de Enfermería, Fisioterapia y Podología, Universidad Complutense de Madrid. Madrid, Spain.; VIII DT, MSc, PhD. Senior Professor, Facultad de Enfermería, Fisioterapia y Podología, Universidad Complutense de Madrid. Madrid, Spain.

**Keywords:** Muscle stretching exercises, Postural balance, Exercise, Ankle, Stabilometry, Platform, Motor control, Continuous passive stretching, Ankle plantar flexor stretching

## Abstract

**BACKGROUND:**

There are no studies on long-term bilateral calf stretching in relation to balance and plantar pressure.

**OBJECTIVES:**

To demonstrate that there is better control of posture and pressures after continuous stretching of the posterior calf muscles.

**DESIGN AND SETTING:**

Pre and post-intervention study conducted in a private clinic.

**METHODS:**

We measured static footprints and stabilometry before and after continuous passive plantar flexor stretching of duration 120 seconds, among 24 healthy subjects.

**RESULTS:**

We found differences in Y displacement with eyes closed (P = 0.010), but not among other variables with eyes closed: X displacement (P = 0.263); surface (P = 0.940); laterolateral speed displacement (P = 0.279); and anteroposterior speed displacement (P = 0.914). There were also no differences in eyes-open variables: X displacement (P = 0.341); Y displacement (P = 0.491); surface (P = 0.167); laterolateral speed displacement (P = 0.852); and anteroposterior speed displacement (P = 0.079). The plantar pressures in the heel (maximum pressure, P = 0.048; mean pressure, P = 0.001) and in the midfoot (maximum pressure, P = 0.004; mean pressure, P = 0.004) were reduced, but not in the forefoot (maximum pressure, P = 0.391; mean pressure, P = 0.225). The surface became larger in the forefoot (P = 0.000) and midfoot (P = 0.021).

**CONCLUSIONS:**

Continuous static stretching of plantar flexors for 120 seconds improved stance balance and reduced plantar pressures (maximum and mean) in the rearfoot and midfoot. It also increased the surface in the midfoot and forefoot.

**TRIAL REGISTRATION:**

at clinicaltrials.gov, under the number NTC03743168.

## INTRODUCTION

Maintaining balance while standing requires constant regulation because the human body is unstable in a static standing position. Balance is regulated through constant adjustment of the ankle torque. This adjustment results from a combination of peripheral reflexes, intrinsic properties of the ankle tissues and regulation from the peripheral and central nervous systems.^1^

It has been recognized that physical performance and the risk of injury can be conditioned through exercises done prior to physical work.^1^ Among these exercises, stretching should be done 15 minutes before the start of the activity in order to benefit from its effects; in fact, just 15 minutes before is when the greatest benefit occurs.^1^ Stretching of the ankle flexor muscles can help to improve muscle functionality in different pathological conditions of the foot and ankle.^2,3,4,5,6,7^

In healthy people, continuous stretching for 30, 60 and 120 seconds was found to improve the range of motion immediately afterwards.^8^ It also lowered gastrocnemius muscle oxygenation levels,^9^ and decreased sports performance when the duration of stretching was 120 seconds or more,^8^ because post-stretch torque loss is associated with decreased central drive.^10^

Lower-extremity muscle strength has proven to be a determining factor in maintaining optimal balance. Low levels of muscle strength have been linked to loss of balance among elderly people.^8^ Continuous prolonged stretching over time produces a decrease in strength after 60 seconds of duration.^10^

Measurement of the movement of the center of pressure (COP) while the subject is standing is one of the usual measurements that have been used in different studies to check postural balance, as it has proven to be reliable.^12^ Within neuromuscular management of the pressure center to regulate balance, the plantar flexor muscles are of decisive importance.^13^ They are capable of adjusting the anteroposterior movement of the COP, which combined with the action of the invertor and evertor muscles (which control the lateral deviation of the COP) are responsible for adjustment of the COP through movement of the ankle.^14^

A few studies in the literature have examined the effects of stretching exercises on balance capacity.^15–19^ Behm et al.^15^ found that static stretching exercises had a negative effect on balance. Lima et al.^15^ studied the acute effects of unilateral static stretching of the ankle plantar flexor on the COP during a single-leg balance task and found that it had a negative effect.^15^ Costa et al.^17^ examined the effects of static stretching exercises on dynamic balance capacity. They determined that a static stretching exercise for 15 seconds could have positive effects on balance capacity.

We did not find any correlation between balance while standing and bilateral continuous calf stretching, from reviewing the literature. Therefore, our hypothesis is that there is better stability when short continuous static stretching is performed, due to reduction of muscle stiffness.

## OBJECTIVE

The aim of our study was to demonstrate that there is better control of posture after continuous stretching of the posterior calf muscles for more than 120 seconds because there is less torque and stiffness but more range of motion.

## METHODS

### Subjects

We recruited 24 healthy subjects, comprising 21 females and three males. The sociodemographic characteristics of the sample population are shown in Table 1. The sample size was calculated by means of the G*Power software (Düsseldorf University, Düsseldorf, Germany).

**Table 1.** Sociodemographic characteristics of the sample population (n = 24)

Variable	Mean ± SD	95% CI
Age (years)	32.20 ± 8.08	(28.97-35.44)
Weight (kg)	62.77 ± 9.52	(58.96-66.57)
Height (cm)	166.20 ± 8.43	(162.83-169.58)
BMI (kg/m^2^)	22.71 ± 2.90	(21.55-23.87)
Shoe size*	38.81 ± 2.26	(37.90-39.72)

BMI = body mass index; SD = standard deviation; 95% CI = 95% confidence interval; *European sizes.

We aimed to test for differences in the center of pressure, in the same was as done in a previous study in which the acute effects of unilateral static stretching of the ankle plantar flexor on the COP during a single-leg balance task were investigated. In that study, it was found that the COP area in the anteroposterior direction improved in the stretched limb from before stretching to immediately after stretching, from 1.06 ± 0.24 to 0.87 ± 0.16 (P = 0.015).^15^

To achieve this with statistical 95% confidence, an 80% statistical power analysis (α = 0.05; β = 20%) and two-tailed tests, a total of 18 participants was found to be required. The subjects were recruited for a month, from October 1, 2018, to October 31, 2018. No subjects were lost over the study period.

The eligibility criteria for the subjects were that they needed to be healthy untrained individuals^15^ who had not engaged in flexibility training for at least six months before the study and refrained from such training during the data collection period. The subjects needed to refrain from vigorous exercise and alcohol consumption for 24 hours and from stimulant use (e.g. caffeine) for six hours before testing.^10^ They also needed to have not had any previous surgery on the lower extremities; have no history of injury with residual symptoms in the lower extremities within the last year; have no evidence of a leg-length discrepancy (difference in distance from the anterior superior iliac spine to the superior surface of the most prominent aspect of the medial malleolus) of more than 1 cm; have at least 15 degrees of ankle dorsiflexion;^20^ and have no evidence of balance deficits, as determined through oral questioning regarding falls^15^ and through using the Balance Evaluation Systems Test (BESTest).^21^

The demographic data of the study participants were as follows: 32.2 ± 8.0 years old; 166.20 ± 8.43 cm height; and 62.77 ± 9.52 kg weight. All the demographic data are shown in Table 1.

All of the subjects were voluntary participants.^22^ The Ethics Committee of A Coruña University approved the study (protocol number: CEIC 28/2016; date: November 28, 2016), and all subjects gave their written informed consent before participating in this research. The ethical standards for human experimentation were in conformity with the Helsinki Declaration. This study was registered at clinicaltrials.gov, under the number NCT03743168.

All measurements were performed at the same hour of the day, between 9 and 11 AM.^15^ First, a clinician confirmed the inclusion and exclusion criteria of each subject and performed a baseline balance evaluation.^21^

The protocol consisted of the following:

- A pre-stretching evaluation.
- An ankle plantar flexor static-stretching protocol. The stretching position consisted of a weight-bearing static stretch: the subject stepped up onto a raised platform and placed the forefoot of both feet on the edge of the platform, dropped both heels off the platform almost to the ground without making contact with it, and held that position. There was one set of continuous stretch,^10^ consisting of 120 seconds of static passive stretching of calf plantar flexors, to the point of discomfort,^15,16^ which was maintained throughout the stretching.^23^ The intensity required was 70%-90% of the point of discomfort (POD),^15^ where 0 = “no stretch discomfort at all” and 100% = “the maximum imaginable stretch discomfort”.^24^ All subjects were asked to assess their POD intensity during all stretches, by a clinician.^15^
- An immediate post-stretching evaluation,^15^ on the same day.

The pre and post-stretching evaluations were performed in a private clinic, which the subjects attended for the purposes of physiotherapy review treatment. During these evaluations, the subjects were instructed to stand barefoot on a force platform.^22^ In preparation for this: the subjects were asked to take up a double-limb stance with placement of their feet on the platform at equal distances from the midline,^25^ and set at 30 degrees to the midline.^26^ The upper limbs were kept loosely alongside the body during all examinations.^27^ The subjects were instructed to stand as still as possible, with their eyes open, while concentrating on a point about two meters away, at eye level.^15^ The subjects stood on the same surfaces with eyes open (EO) or eyes closed (EC).^22^

Two tests (EO and EC) were performed, each of 30 seconds in duration,^15,22,27^ and the order of the EO and EC conditions was randomized across the subjects.^22^ This randomization of the order of the tests was implemented using a bag from which the subject extracted a piece of paper that stated which test was to be performed. Foot plantar pressure was measured during this bipodal standing, with placement of the patient’s feet on the platform, before and after stretching. We did two trials on each condition (EO and EC) and the foot area was divided into 3 bilateral areas: bilateral rearfoot, bilateral midfoot, bilateral forefoot.

### Variables

The stabilometry measurements consisted of the displacement of the centers of pressure in X and Y with open and closed eyes,^22^ the COP area and the COP speed in the anteroposterior (a-p) and mediolateral directions.^22^

Ground reaction forces and moments were recorded and digitized using the Podoprint system (Medicapteurs; Balma, France) with 2,304 sensors in an area of 400 mm x 400 mm, and an acquisition frequency of 200 Hz, with an auto-calibrated system for any use.

### Statistical analysis

All data were explored for normality using the Shapiro-Wilks test because the sample size was less than 30 subjects. From this, the data were considered to be normally distributed if P > 0.05. A descriptive statistical analysis was performed using means ± standard deviations (SD) and 95% confidence intervals. For each intrasession trial, the intraclass correlation coefficient (ICC) was used to evaluate the reliability of each parameter. To interpret ICC values, we used benchmarks as proposed by Landis and Koch:^28^ 0.20 or less, slight agreement; 0.21 to 0.40, fair; 0.41 to 0.60, moderate; 0.61 to 0.80, substantial; and 0.81 or greater, almost perfect.

Standard errors of the mean (SEM) were calculated to measure the range of error of each parameter. The SEM was calculated between sessions, from the ICCs and SDs. SEM = s_x.√(1- r_xx); where s_x is the standard deviation of the observed set of test scores, and r_xx is the reliability coefficient for these data, which in this case was considered using the ICC.

Also, the mean value from two measurements on each variable was used. The Wilcoxon signed rank test was performed to test for any differences in nonparametric variables and paired t tests were used for parametric variables.

Lastly, values of normality (VN) were defined for the sample, for all variables. These were obtained from the formula VN = mean +/-1.96 * SD. From the result from each variable, VN was used to calculate the 95% confidence interval. P-values < 0.05 with a 95% confidence interval were considered statistically significant for all tests. The SPSS for Windows software, version 20.0, was used (SPSS Inc., Chicago, Illinois, United States).

The analysis on the intrasession reliability of the variables studied and the values of normality for the total population are presented in Table 2.

**Table 2.** Analysis on intrasession reliability of the variables studied and values of normality in the total population (n = 24)

Variable	Pretest (n = 24)			Posttest (n = 24)		
	ICC (95% CI)	SEM	Values of normality 95% CI	ICC (95% CI)	SEM	Values of normality 95% CI
Rearfoot maximum pressure (kPa)	0.822 (0.549-0.929)	10.24	59.97-155.17	0.990 (0.976-0.996)	1.96	62.13-139.13
Rearfoot mean pressure (kPa)	0.988 (0.970-0.995)	0.62	29.81-52.31	0.981 (0.952-0.992)	0.79	26.16-48.66
Rearfoot surface (cm^2^)	0.992 (0.982-0.997)	0.94	925.69-884.14	0.995 (0.989-0.998)	0.90	1097.99-1047.69
Midfoot maximum pressure (kPa)	0.996 (0.990-0.998)	0.75	-10.82-35.90	0.812 (0.526-0.926)	6.58	-12.45-47.05
Midfoot mean pressure (kPa)	0.994 (0.984-0.997)	0.44	-5.13-17.59	0.910 (0.771-0.964)	1.90	-4.18-20.66
Midfoot surface (cm^2^)	0.991 (0.979-0.996)	1.87	383.13-305.51	0.995 (0.989-0.998)	1.33	413.97-340.04
Forefoot maximum pressure (kPa)	0.972 (0.930-0.989)	0.82	49.16-90.94	0.743 (0.349-0.898)	8.16	41.15-103.93
Forefoot mean pressure (kPa)	0.969 (0.922-0.988)	0.48	20.01-30.75	0.496 (-0.274-0.800)	5.08	12.82-40.88
Forefoot surface (cm^2^)	0.987 (0.969-0.994)	1.55	1269.83-1216.52	0.986 (0.967-0.994)	1.94	1379.85-1309.96
X displacement with eyes open (mm)	0.990 (0.974- 0.996)	0.43	0.21-17.41	0.986 (0.965-0.995)	0.59	-2.15-17.63
Y displacement with eyes open (mm)	0.945 (0.860- 0.978)	1.98	4.07-37.19	0.985 (0.961-0.994)	1.33	-2- 40.76
Surface with eyes open (mm^2^)	0.986 (0.964-0.994)	0.79	5.17-29.13	0.986 (0.965- 0.995)	1.03	5.17-29.13
Mean speed of laterolateral displacement with eyes open (mm/s)	0.865 (0.659-0.947)	0.10	0.65-1.73	0.920 (-1.294-.641)	0.20	−0.09-2.69
Mean speed of anteroposterior displacement with eyes open (mm/s)	0.785 (0.457- 0.915)	0.09	0.49-1.47	0.919 (0.794-0.968)	0.11	0.09-1.73
X displacement with eyes closed (mm)	0.950 (0.874-0.980)	1.04	1.31-17.03	0.985 (0.963-0.994)	0.52	−1.73-15.07
Y displacement with eyes closed (mm)	0.972 (0.928-0.989)	1.63	4.68-40.66	0.982 (0.955-0.993)	1.28	−0.91-36.71
Surface with eyes closed (mm^2^)	0.985 (0.963-0.994)	1.33	2.30-21.28	0.966 (0.915-0.987)	2.58	−3.93-50.95
Mean speed of laterolateral displacement with eyes closed (mm/s)	0.768 (0.415-0.908)	0.11	0.87-1.77	0.851 (0.623-0.941)	0.18	0.34-2.22
Mean speed of anteroposterior displacement with eyes closed (mm/s)	0.853 (0.630-0.942)	0.12	0.62-1.90	0.092 (-1.294-0.641)	0.94	−0.49 -3.39

SD = standard deviation; 95% CI = 95% confidence interval.

## RESULTS

All the variables showed non-normal distribution (P < 0.05).

Stabilometry and static footprint variables before and after bilateral stretching are presented in Table 3. Most of the mean values were similar before and after stretching. The exception was the mean Y displacement variable with eyes closed, which was statistically significantly lower after stretching.

**Table 3.** Stabilometry and static footprint variables before and after bilateral stretching

Variable	Pretest ( n = 24) Mean ± SD (95% CI)	Posttest (n = 24) Mean ± SD (95% CI)	P-value*
Rearfoot maximum pressure (kPa)	107.57 ± 24.29 (96.20-118.94)	100.66 ± 19.63 (91.47-109.85)	0.048
Rearfoot mean pressure (kPa)	41.06 ± 5.74 (38.37-43.75)	37.41 ± 5.74 (34.72-40.10)	0.001
Rearfoot surface (cm^2^)	85.37 ± 10.60 (80.89-89.85)	83.62 ± 12.83 (78.20-89.04)	0.196
Midfoot maximum pressure (kPa)	12.54 ± 11.92 (6.95-18.12)	17.30 ± 15.18 (1019-24.40)	0.004
Midfoot mean pressure (kPa)	6.23 ± 5.80 (3.51-8.94)	8.24 ± 6.34 (5.27-11.21)	0.004
Midfoot surface (cm^2^)	17.39 ± 19.80 (9.03-25.76)	19.99 ± 18.86 (12.03-27.96)	0.021
Forefoot maximum pressure (kPa)	70.05 ± 10.66 (65.06-75.04)	72.54 ± 16.02 (65.04-80.04)	0.391
Forefoot mean pressure (kPa)	25.38 ± 2.74 (24.09-26.66)	26.85 ± 7.16 (23.49-30.20)	0.225
Forefoot surface (cm^2^)	91.41 ± 13.60 (85.67-97.16)	99.50 ± 16.43) (92.56-106.43)	0.000
X displacement with eyes open (mm)	8.81 ± 4.39 (6.75- 10.86)	7.74 ± 5.05 (5.38-10.11)	0.341
Y displacement with eyes open (mm)	20.63 ± 8.45 (16.67-24.59)	19.38 ± 10.91 (14.27-24.49)	0.490
Surface with eyes open (mm^2^)	10.02 ± 6.72 (6.88-13.17)	11.98 ± 8.75 (7.88-16.07)	0.167
Mean speed of laterolateral displacement with eyes open (mm/s)	1.19 ± 0.28 (1.05-1.32)	1.30 ± 0.71 (0.96-1.63)	0.852
Mean speed of anteroposterior displacement with eyes open (mm/s)	0.98 ± 0.25 (0.86-1.10)	0.91 ± 0.42 (0.71-1.11)	0.079
X displacement with eyes closed (mm)	7.86 ± 4.68 (5.67-10.05)	6.67 ± 4.29 (4.66-8.68)	0.263
Y displacement with eyes closed (mm)	22.67 ± 9.18 (18.37-26.97)	17.90 ± 9.60 (13.41-24.03)	0.010
Surface with eyes closed (mm^2^)	23.58 ± 10.86 (18.50-28.66)	23.51 ± 14.00 (16.95-30.06)	0.940
Mean speed of laterolateral displacement with eyes closed (mm/s)	1.32 ± 0.23 (1.21-1.43)	1.28 ± 0.48 (1.05-1.51)	0.279
Mean speed of anteroposterior displacement with eyes closed (mm/s)	1.26 ± 0.33 (1.10-1.42)	1.45 ± 0.99 (0.99-1.91)	0.914

SD = standard deviation; 95% CI = 95% confidence interval; *Wilcoxon test: P-value < 0.05 taken to be significant.

**Figure 1.**
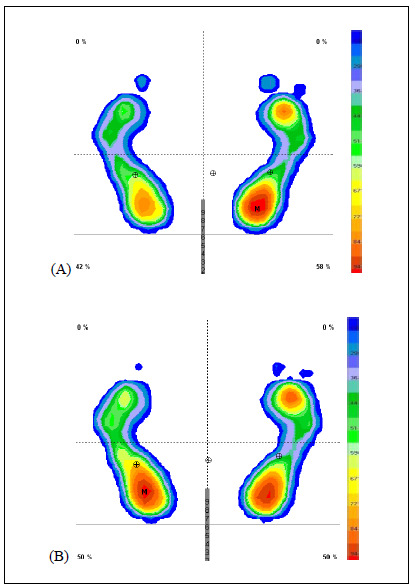
Distribution of pressure in static footprint for a representative subject before stretching (A); and distribution of pressure in static footprint for a representative subject after stretching (B). The scale at the right indicates pressure (g/cm^2^).

After stretching, the plantar pressures (maximum and mean) in the heel, midfoot and forefoot were lower. The surface in the midfoot and forefoot became larger. A static footprint for a representative subject before and after stretching is shown in Figure 1.

## DISCUSSION

The aim of this intervention study was to analyze changes to balance and footprint variables immediately after 120 seconds of continuous stretching of plantar flexors. After stretching, the plantar pressures (maximum and mean) in the rearfoot and midfoot were reduced. The forefoot pressures did not change. The surface variable in the midfoot and forefoot became larger. We believe that the increase in the surface of the midfoot and forefoot contributed to the decrease in the pressures in the rearfoot and midfoot.

Limitation to dorsiflexion of the ankle in cases of plantar forefoot ulceration has been shown to be beneficial after lengthening of the Achilles tendon, with regard to ulcer recurrence, due to the reduction of plantar pressures.^29^ Interestingly, with 120 seconds of continuous plantar flexor stretching, we observed reduced pressures in the forefoot, in feet without an equinus condition. Thus, healthy people may avoid having foot pathological conditions in which the symptoms consist of increased pressure, such as metatarsalgia or hallux abductor valgus.^30^ Additional studies are needed in order to ascertain the duration of the effect of the pressure reduction.

The balance variables evaluated with eyes open before and after stretching did not show any significant differences. On the other hand, with eyes closed, the Y displacement became significantly lower after stretching. This finding was concordant data obtained in other studies on stance balance. The ankle and gastrocnemius muscle are activated through the anteroposterior movement of the center of pressure during bipodal standing. For mediolateral movement, a separate hip load/unload strategy implemented by the hip abductors/adductors forms a dominant defense for standing with feet side-by-side.^31^ Logically, our intervention on the gastrocnemius affected only the posterior displacement.

Comparing our results with previous research, the types and protocols of stretching differed in all studies, and the results too. Behm et al.^16^ found that static stretching exercises had a negative effect on balance, using an intermittent stretching protocol. Morrin and Redding^18^ compared the effects of three different stretching protocols. The results showed that combination stretching gave rise to significantly enhanced balance and vertical jump height scores than those from static stretching. Their stretching protocol was performed on the hamstring muscles.

It needs to be considered that, depending on the muscle studied, different acute effects from static stretching on muscle-tendon mechanics can be observed.^31^ Chatzopoulos et al.^19^ found that static stretching had a negative effect on dynamic balance,^32^ in a protocol consisting of three minutes of jogging followed by seven minutes of static stretching. Lima et al.^15^ tested a protocol for unilateral static discontinuous stretching of ankle plantar flexors and analyzed the open-eye condition in relation to unilateral balance.^15^ In contrast, we analyzed a bipodal stance, which requires low-intensity contraction of the triceps surae, compared with unipodal balance. Lim et al.^33^ indicated that static stretching exercises had no effect on static balance.

There is a consensus regarding the dose-effect relationship of the duration of stretching.^34,35^ Young et al.^34^ studied the effects of the volume and intensity of warm-up static stretching on explosive force production and range of motion (ROM) of the plantar flexors. Two minutes of stretching at 90% intensity had no significant influence on muscle function in these variables.

We believe that the negative effects on static balance that were seen in previous studies differed from ours due to the type of stretching and its duration, and also regarding the measurement with eyes closed. In this regard, Costa et al.^17^ determined that static stretching exercises with durations of 15 and 45 seconds could have positive effects on dynamic balance capacity.

The physiological changes that can occur in short-duration medium-intensity static stretching on the osteotendinous reflex and Golgi reflex are probably different from the changes seen through longer-duration high-intensity stretching. Therefore, the input to the central nervous system from long and high-intensity stretching, and the subsequent reorganization of the central nervous system, ought to be different from the input from short and medium-intensity stretching.^17^

We consider that medium-intensity stretching with a duration of 120 seconds has an effect consisting of proprioceptive improvement and believe that this was the cause of the results from our study. Any stimulus that increases proprioception shows its importance when a proprioceptive route as important as the visual route is then removed. However, additional studies are needed in order to acquire evidence regarding the physiological causes of the findings and their possible applications in sports and rehabilitation.

One limitation of this study was that the sample of subjects was not equitable in gender terms. Nonetheless, it is true that most studies on stretching have not reflected equity between men and women since this factor is not considered to affect the result.^8,10^

The present study showed better stance balance and lower pressures in the rearfoot and midfoot. This was probably due to the increased surface area in the forefoot. This implies that there is a need for stretching among subjects with previous histories of lesions due to high midfoot and rearfoot pressures, especially in barefoot exercises and in sports that imply a need for greater static balance against force, such as archery.

## CONCLUSIONS

This study demonstrated that continuous static stretching of plantar flexor muscles for 120 seconds improved stance balance and reduced the plantar pressures (maximum and mean) in the rearfoot and midfoot. Stretching increased the surface variable in the midfoot and forefoot.
